# Supraglottic airway versus endotracheal tube during interventional pulmonary procedures – a retrospective study

**DOI:** 10.1186/s12871-019-0872-x

**Published:** 2019-10-31

**Authors:** Kyle M. Behrens, Richard E. Galgon

**Affiliations:** 1 Chicago Medical School, Rosalind Franklin University of Medicine and Science, 3333 Green Bay Road, North Chicago, IL 60064 USA; 2 Department of Anesthesiology, University of Wisconsin School of Medicine and Public Health, 600 Highland Ave., B6/319, Madison, WI 53792 USA

**Keywords:** Supraglottic airway, Endotracheal tube, Airway maintenance, Interventional pulmonology, Neuromuscular blocking

## Abstract

**Background:**

As the field of interventional pulmonology (IP) expands, anesthesia services are increasingly being utilized when complex procedures of longer duration are performed on sicker patients with high risk co-morbidities and lung pathology. Yet, evidence on the optimal anesthetic management for these patients remains lacking. Our aim was to characterize the airway management and, secondarily anesthetic maintenance patterns used for IP procedures at our institution.

**Methods:**

From 2894 identified encounters, charts of 783 patients undergoing an IP procedure with general anesthesia over a 5-year period, employing an endotracheal tube (ETT) or a supraglottic airway (SGA) for airway maintenance, were identified and reviewed after exclusions. Patients posted for a concurrent thoracic surgical procedure and those already intubated at presentation were excluded. Baseline patient demographics, procedure, proceduralist type, anesthesia maintenance modality, neuromuscular blocking drug (NMBD) use, and airway management characteristics were extracted and analyzed.

**Results:**

Inhaled general anesthesia with an ETT for airway maintenance was most commonly employed; however, SGAs were used in one-third of patients with a very low conversion rate (0.4%), and their use was associated with a significant reduction in NMBD use.

**Conclusions:**

In this large series of patients receiving general anesthesia for IP procedures, inhaled anesthetic agents and ETTs were favored. However, in appropriately selected patients, SGA use was effective for airway maintenance and allowed for a reduction in NMBD use, which may have implications in this patient population who may have an increased risk for pulmonary complications and warrants further investigation.

## Background

The field of interventional pulmonology (IP) is rapidly expanding as new technologies and techniques are invented with nearly 500,000 bronchoscopies being performed in the United States each year [1, 2]. Sicker patients with high risk co-morbidities and lung pathology are now able to undergo less invasive procedures resulting in shorter hospital stays. It is common for patients with less co-morbidities to be managed effectively using conscious sedation during these procedures, which can even be administered/directed by the interventionalist. However, many US and European medical centers have made it standard practice to have an anesthesiologist provide either sedation or general anesthesia to selected high risk patients undergoing IP procedures to safely manage them [3, 4].

It has been nearly 35 years since supraglottic airways (SGAs) have been released for anesthetic practice; however, SGAs have not been the standard of care to facilitate IP procedures due to the increased potential for dislodgement and less airway control compared to endotracheal tube (ETT). Over time, anesthesia providers and interventionalist with experience using SGAs have allowed their scope to be advanced. Advantages of using SGAs (compared to ETT) include (1) quicker and easier placement, (2) reduction in neuromuscular blocking drug (NMBD) usage, residual paralysis, hemodynamic variability, anesthetic requirement for device placement, emergence coughing, and laryngeal and subglottic trauma, and (3) preservation of laryngeal competence and mucociliary function [5]. In a report in 2016, Arevalo-Ludena et al reported observing no difference in leakage between SGAs and ETT during bronchoscopic lung volume reduction procedures [6]. However, data on the use of SGAs for other procedures remains lacking.

Over the course of the last five to ten years, the scope of SGA usage at our institution for IP procedures has grown, particularly because the use of an SGA for IP procedures affords versatility over use of an ETT in selected patients by providing the ability to (1) perform a complete airway exam, including visualization of glottic structures, (2) biopsy more proximal lymph nodes, and (3) manipulate endobronchial devices more easily through the airway conduit. The purpose of our study was to characterize the use of SGA versus ETT, and secondarily anesthetic maintenance patterns, during IP procedures at our institution.

## Methods

For this study, after Institutional Review Board review and exemption from informed consent, we performed a retrospective chart review. Consecutive patients who underwent an IP procedure at our institution and were cared for by an anesthesia provider during the period of April 15, 2008 through April 14, 2013 (5 years) were identified and included using anesthesia departmental billing records. A priori exclusion criteria included patients who underwent airway management by facemask, rigid bronchoscopy, or jet ventilation, those who underwent a concurrent surgical procedure and those already intubated at presentation. The primary analysis was focused on comparing the use of an SGA versus ETT for airway maintenance, and the need to convert use of an SGA to an ETT during the procedure (i.e., SGA failure). SGA failure was defined as a need to place an ETT during the procedure secondary to poor airway seal performance. SGA failure did not include incidence of airway placement failure at the initial start of the procedure. The determination to use an SGA or ETT was made by the attending anesthesiologist at the time of the procedure. Data including (1) patient characteristics (e.g., age, gender, weight, height, co-morbid diseases, such as diabetes mellitus, gastroesophageal reflux disease, hiatal hernia, or a history of neck radiation, known airway management difficulties, and airway exam findings), (2) procedure type (e.g., flexible bronchoscopy, endobronchial ultrasound, endobronchial tumor debulking, super dimensional bronchoscopy, etc.), and (3) anesthetic management characteristics (e.g., intravenous versus inhalational agent use, ventilation mode, and NMBD), were defined a priori and extracted for secondary exploratory analyses. NMBD use was recorded if an intermediate acting NMBD was administered or re-dosed during the procedure. If a short acting NMBD (succinylcholine) was given to facilitate airway management device placement and no other NMBDs were administered, NMBD use was not counted. Descriptive statistics (mean (SD) and percent) were used to characterize group data. Intergroup comparisons (SGA versus ETT) were performed using t-tests for continuous data and chi square or Fisher’s exact tests for categorical data, using GraphPad Prism (Version 5.0, GraphPad Software Inc., La Jolla, CA). Statistical significance was considered at a p-level <  0.05.

## Results

**Fig. 1:**
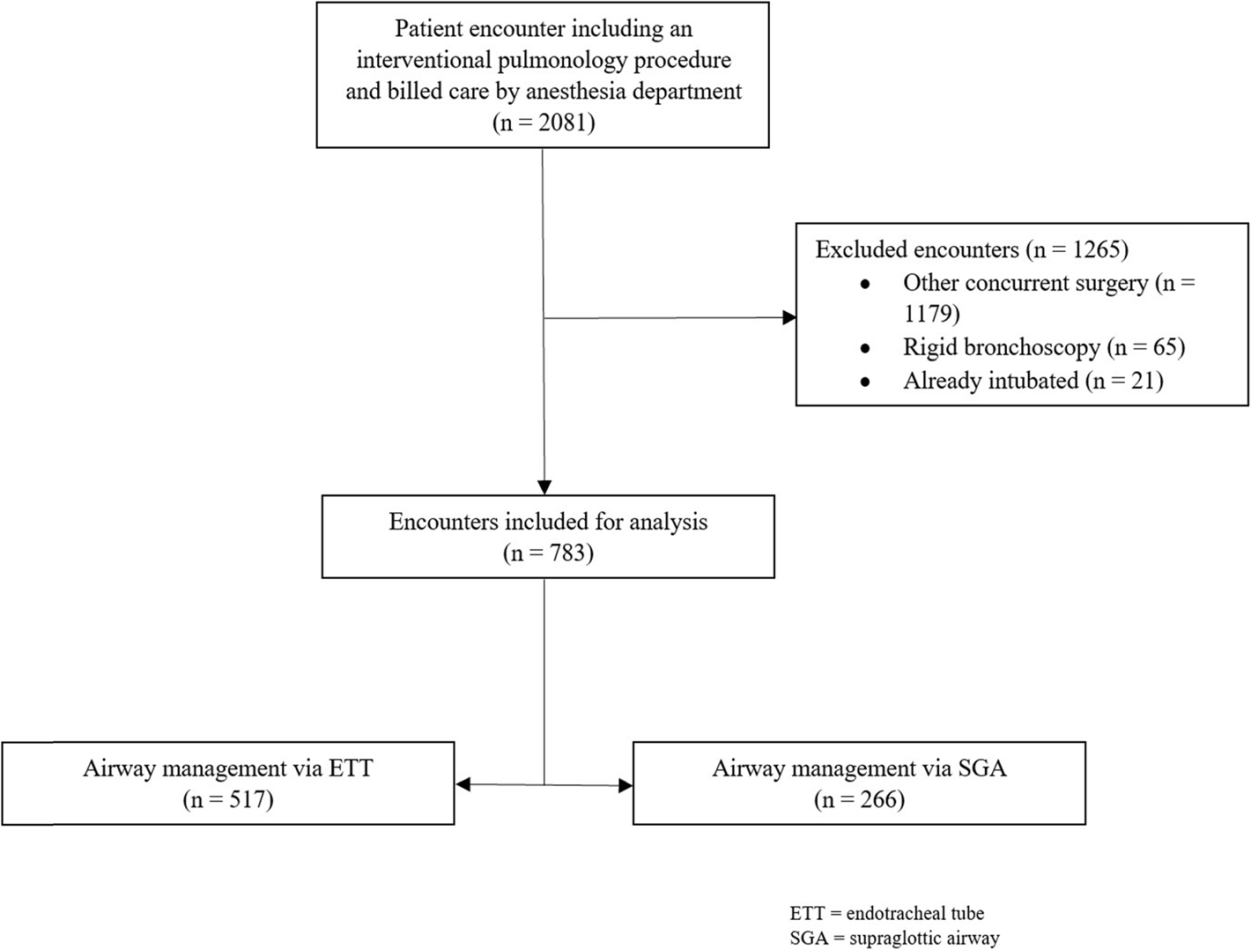
Study Flow Diagram

During the study period, 2081 encounters meeting the study inclusion criteria were identified. From these, 783 records were analyzed after exclusions (see Fig. 1 for study flow diagram). Overall, 39.3, 57.3, and 3.4% of the patients underwent flexible diagnostic bronchoscopy with or without transtracheal fine needle aspiration biopsy, endobronchial ultrasound (EBUS) guided transtracheal fine needle aspiration biopsy, and tracheal and/or bronchial laser debulking and/or placement procedures, respectively. Interventional pulmonologists performed these procedures 72.2% of the time, while a thoracic surgeon performed 27.8% of the procedures. General anesthesia was maintained using inhalational agents in the majority of cases (85.7% versus 14.3% for intravenous agents). Five hundred seventeen patients were managed using an ETT, while 266 patients were managed using an SGA, providing study groups for intergroup comparisons.

For the intergroup comparisons, baseline patient demographics and device performance characteristics are shown in Table 1. There were no significant differences noted between the comparison groups amongst the baseline characteristics, including characteristics that might suggest an increase risk for pulmonary aspiration, poor SGA fit, or difficult airway management. Endotracheal tube was preferred for flexible bronchoscopy procedures, while SGA was preferred for endobronchial ultrasound guided diagnostic procedures. Tracheal and/or bronchial laser debulking and/or stent procedures showed no preference between airway device usage. With respect to device performance, SGA conversion rate (to ETT) was 0.4% [95% CI: 0.0, 2.3%]. SGA versus ETT use was also associated with a significant reduction in NMBD administration (9.0% [6.1, 13.1%] versus 78.3% [74.6, 81.7%]).

**Table 1:** Baseline Characteristics and Device Performance Comparisons

	ETT	SGA	
	(*n* = 517)	(*n* = 266)	*p*-value^‡^
Age, yrs	61 (13)	62 (13)	0.43
Gender, n (%)			
Male	294 (56.9%)	143 (53.8%)	0.45
Female	223 (43.1%)	123 (46.2%)	
ASA, n (%)			
1	2 (0.4%)	2 (0.8%)	0.77
2	187 (36.2%)	91 (34.2%)	
3	300 (58.0%)	161 (60.5%)	
4	28 (5.4%)	12 (4.5%)	
Co-morbidities, n (%)			
DM	81 (15.7%)	39 (14.7%)	0.75
GERD	155 (30.0%)	88 (33.1%)	0.37
OSA	39 (7.5%)	27 (10.2%)	0.22
Known difficult intubation	10 (1.9%)	6 (2.3%)	0.42
BMI, kg∙m^−2^	28 (6)	28 (7)	0.55
Mallampati score, n (%)			
I	115 (32.1%)	62 (28.6%)	0.55
II	199 (55.6%)	123 (56.7%)	
III	44 (12.3%)	27 (12.4%)	
IV	0 (0%)	5 (2.3%)	
Mouth opening, n (%)			
< 4 cm	27 (6.5%)	20 (9.2%)	0.59
> 4 cm	388 (93.5%)	211 (90.8%)	
Upper lip bite test, n (%)			
Achieved	184 (94.4%)	103 (96.3%)	0.34
Not Achieved	11 (5.6%)	4 (3.7%)	
Thyromental distance, n (%)			
< 6 cm	42 (10.6%)	25 (11.5%)	0.79
> 6 cm	355 (89.4%)	193 (88.5%)	
Neck range of motion, n (%)			
Full	271 (79.2%)	149 (76.8%)	0.51
Limited	70 (20.8%)	45 (23.2%)	< 0.0001
Procedure type, n (%)			
Flexible bronchoscopy y	233 (45%)	74 (28%)	< 0.0001
Endobronchial ultrasound	263 (51%)	185 (70%)	0.42
Tracheal/bronchial laser/stent	20 (4%)	7 (3%)	
Anesthetic type, n (%)			
Inhalational	438 (84.7%)	233 (87.6%)	0.33
TIVA	79 (15.3%)	33 (12.4%)	
^†^Neuromuscular blocking drug use, n (%)	405 (78.3%)	24 (9.0%)	< 0.0001
Failed primary airway, n (%)	1 (0.4%)		

Data are mean (SD) unless otherwise noted. *ASA* American Society of Anesthesiologists, *DM* diabetes mellitus, *GERD* gastroesophageal reflux disease, *OSA* obstructive sleep apnea, *BMI* body mass index, *TIVA* total intravenous anesthesia, *ETT* endotracheal tube, *SGA* supraglottic airway. ^†^Includes intermediate or long-acting neuromuscular blocking drugs; excludes succinylcholine used for airway placement. ^‡^ Intergroup comparisons were performed using t-tests and chi square or Fisher’s exact tests for continuous and categorical data, respectively. A *p*-value < 0.05 was considered statistically significant

## Discussion

The main finding of this study is that usage of SGAs for IP procedures can be highly successful with a low conversion rate to ETT when used in appropriately selected patients. Secondarily, when SGAs are used for IP procedures in our institution, the avoidance of NMBDs and the potential for consequent residual paralysis in a patient population that may have significant underlying pulmonary co-morbidities is achieved.

With the advancement of therapeutic and interventional procedures in the field of IP in recent years, there has been limited literature showing the usage and successfulness of SGAs compared to ETTs in anesthetized patients. Previously, Du Plessis et al found that during 140 adult patients undergoing fiberoptic bronchoscopy with general anesthesia using an SGA, only one patient required tracheal intubation due to laryngospasms, a conversion rate of 0.7% [7]. In a different retrospective study of 200 patients having underwent awake diagnostic bronchoscopies, use of an SGA facilitated successful bronchoscopies in every patient except one, where device placement was not tolerated [8]. Finally, in a recent publication by Schmutz et al*,* an SGA failure rate was found to be 3.1% in 132 patients that underwent transbronchial lung cryobiopsy, which the authors attributed to impossible placement of SGA (*n* = 1), high oropharyngeal leakage (n = 1), massive endobronchial bleeding (n = 1), and acute right heart failure requiring resuscitation (n = 1) [9]. In our study, which includes a broader range of IP procedures, we found similar success in the use of SGAs for airway maintenance during general anesthesia.

Previous authors have discussed some advantages of SGA use during fiberoptic bronchoscopy under general anesthesia [10]. Beyond the use of decreased NMBDs use, advantages of using an SGA over an ETT for IP procedures based on clinical experience includes easier placement, examination of glottis and upper trachea, easier device placement including bronchoscope, lighter depth of anesthesia requirement, and smoother emergence. Additionally, based on our experience, we suggest several ideal features of SGAs for IP procedures. First, an SGA with a good oropharyngeal seal and stable fit enables positive pressure ventilation when needed at a higher airway pressure and may be less prone to dislodgement. Second, an SGA with a relatively short, straight, and large internal diameter air tube reduces resistance to bronchoscopic device movements, facilitating the IP procedure. Third, an SGA with a built-in bite block protects the bronchoscopic equipment from damage due to potential patient biting during the procedure. Finally, an SGA with a gastric channel enables decompression of a patient’s stomach if desired, and also provides a means to reseat a dislodged SGA over an orogastric tube if left in situ.

Although our study provides evidence to support the use of SGAs for IP procedures, it is not without limitations. First, several different types of SGAs (Ambu® AuraStratight™, Ambu® AuraFlex™, LMA® Supreme™, LMA® ProSeal™, i-gel®, and air-Q®) were in use in our institution during the study period, and the specific SGA used for each case was not routinely recorded in the chart. Therefore, we cannot ascribe any success to a particular device. During the last two years of the study period, however, it is known that the i-gel® (over the Ambu® AuraStraight™) started to be used routinely for IP procedures, and continues to enjoy predominant use today, as it incorporates many of the ideal features noted above. Second, our study is a retrospective study, which can suffer from selection biases, variations in intra-operative management techniques, and missing data specifics, amongst others. It can be noted that at the time of these procedures patient characteristics, previous anesthesia and surgical experience, and local culture may have led to the anesthesiologist preferentially using one airway device over the others. The results of our study also do not show a correlation between airway device selection and general anesthesia maintenance (i.e. inhalational versus total intravenous anesthesia). Although prior reports indicate some advantages to total intravenous anesthesia, the majority of IP procedures at our institution continue to be performed using inhalation anesthesia due to attending anesthesiologist preferences [11]. Finally, two main modes of ventilation were used during the study period. For patients in the ETT group, the most common mode of ventilation was mandatory volume control ventilation (VCV) (56%), followed by mandatory pressure control ventilation (PCV) (39%). In the SGA group, the most common primary mode of ventilation was mandatory PCV (50%), followed by mandatory VCV (22%). Less commonly in both groups, patients were intermittently managed with other modes of ventilation. Specific ventilator parameters were not recorded in the database. Generally during the procedure, a standard bronchoscope with a sideport connection was placed on the proximal end of airway device facilitating passage of the bronchoscope into the patient’s trachea and pulmonary tree. Patient ventilation and oxygenation was monitored using end-tidal carbon dioxide and pulse oximetry. If inadequate ventilation or oxygenation was a concern, the anesthesiologist communicated to the interventionalist to halt the procedure and withdrawal the bronchoscope to allow for collection of adequate monitor data. Adjustments in oxygen delivery and/or ventilation parameters were then made. Operating room pollution from inhalation agents was controlled using a standard gas scavenger system. Anesthesia providers who were uncomfortable with the performance of the SGA during the procedure due to air leak, exchanged the device for an ETT. This occurred in 0.4% of patients whose airways were managed with an SGA. We would like to acknowledge that high frequency jet ventilation has been commonly used during interventional pulmonology procedures at other institutions, however, this was not a mode of ventilation available at our institution and was not used during the study period. Nonetheless, despite the limitations of our study, our study is the largest study on the topic, and we believe the results provide support for the use of SGAs for airway maintenance during IP procedures, particularly given the limited availability of other data.

## Conclusions

In summary, our study has shown ETT are more commonly used in practice for IP procedures, but evidence of successful SGA usage and the potential reduction in NMBD administration can be achieved in appropriate patient populations.

## Abbreviations

ETT: Endotracheal tube
IP: Interventional pulmonology
NMBD: Neuromuscular blocking drugs
PCV: Pressure control ventilation
SGA: Supraglottic airway
VCV: Volume control ventilation
